# Initiating and Maintaining ADHD Medications in Islington CAMHS 2023

**DOI:** 10.1192/bjo.2025.10548

**Published:** 2025-06-20

**Authors:** Emmanuel Ameh

**Affiliations:** North London Foundation NHS Trust, London, United Kingdom

## Abstract

**Aims:** To evaluate and improve the adherence to NICE guidelines in recording physical health parameters during the initiation and maintenance of ADHD medications.

**Methods:** Retrospective data was collected from electronic medical records (RIO) of patients who were initiated and maintained on ADHD medication, referred to Islington CAMHS in 2023. Collected data was stored and analysed using Microsoft Excel. Inclusion criteria:

1. All the patients with ADHD referred to Islington CAMHS in 2023 for initiation of medication were sampled.

Exclusion criteria:

1. Patients not started on medication.

2. Patients who had previously been on medication.

3. Patients who had medication initiated by paediatrician.

4. Patients who had medication initiated by private psychiatrist.

**Results:** A total of 74 patients were identified, 55 males and 19 females.

Pre-medication Physical Health Assessment: The physical health parameters before initiating medication were recorded in 100% of cases, with 96% adhering to the standards outlined by NICE. This indicates a strong adherence to pre-medication assessment protocols.

Medikinet XL was the most prescribed medication for both initiation and maintenance.

Side Effects and Medication Management: Side effects were reported by 22% of patients, with reduced appetite being the most common. Medication was stopped in 4% of cases due to side effects, and 11% required a change in medication. This highlights the importance of ongoing monitoring and the need for flexibility in treatment plans to address side effects promptly.

Standard Monitoring Compliance: The monitoring of physical health parameters during medication maintenance also met the standards in 100% of cases, underscoring a consistent approach to ongoing patient care.

Comorbidities: The audit identified patients with psychiatric comorbidities, such as Autism Spectrum Condition (47%), Tourette’s (3%), Anxiety (3%), and Dyslexia (1%).

**Conclusion:** The audit highlighted the importance of maintaining high standards in ADHD medication management and suggested areas for further improvement, such as the documentation of rating scales to objectively monitor medication effectiveness. Based on the audit results, an ADHD clinic proforma was developed to incorporate all required data points, ensuring comprehensive documentation during clinic appointments.

